# Editorial: Pathogenomics of the Genus *Brucella* and Beyond

**DOI:** 10.3389/fmicb.2021.700734

**Published:** 2021-07-08

**Authors:** Axel Cloeckaert, Michel S. Zygmunt, Holger C. Scholz, Nieves Vizcaino, Adrian M. Whatmore

**Affiliations:** ^1^INRAE, Université de Tours, UMR, ISP, Nouzilly, France; ^2^Centre for Biological Threats and Special Pathogens, Highly Pathogenic Microorganisms (ZBS 2), Robert Koch Institute, Berlin, Germany; ^3^Departamento de Microbiología y Genética, Universidad de Salamanca, Salamanca, Spain; ^4^Department of Bacteriology, Animal and Plant Health Agency, Weybridge, United Kingdom

**Keywords:** *Brucellaceae*, *Brucella*, *Ochrobactrum*, genetics/genomics, diversity, evolution, cell envelope, virulence

## Introduction

Brucellosis, caused by members of the genus *Brucella*, is an economically important disease in production animals causing abortion and infertility. Human brucellosis is associated with animal reservoirs and transmitted via the food chain or direct contact with diseased animals. This collection of 30 review, opinion and original research articles add to understanding of this group in fundamental areas from emerging genomic approaches and their application, through to knowledge of diverse aspects of lifestyle and pathogenicity.

## *Brucella* Within the Family *Brucellaceae*

One ongoing debate is how *Brucella* should be classified taxonomically, both internally within the genus, and in terms of wider relationships with other members of family *Brucellaceae*. This debate has continued for years, promoted by the emergence of genetically atypical *Brucella* strains distinct from classical *Brucella* species, and the discovery of many new non-*Brucella* members of the family *Brucellaceae*. Two papers in the Research Topic explore genetic relationships within *Brucellaceae* using whole genome sequencing (WGS). Leclercq et al. compared 145 *Brucellaceae* genomes with over 40 others from the wider order *Rhizobiales* to resolve phylogenetic ambiguities. They positioned the entire genus *Brucella* as a single genomic clade within current *Ochrobactrum* species diversity, while also separating *Ochrobactrum* species themselves into two distinct clades. The authors speculate that one species, *Ochrobactrum intermedium*, is undergoing genome reduction that may lead to an animal-associated pathogenic lifestyle rather than a saprophytic lifestyle. This mirrors genome reduction thought to have occurred in the evolution of the highly pathogenic *Brucella* species (Wattam et al., 2014). Ashford et al. focused in depth on the *Brucellaceae* family again showing that *Ochrobactrum*, as defined at the time of writing, is a polyphyletic group splitting into two clades. The study also recognized substantial currently unindexed diversity in *Ochrobactrum* spp. and *Pseudochrobactrum* spp. when non-type strains were included in the analysis.

## Novel *Brucella* Species and Strains

An emerging theme of recent years has been the recognition of new groups of strains expanding known ecological niches and genetic diversity of the genus *Brucella* (Moreno, 2020; Whatmore and Foster, 2021). Guzmán-Verri et al. characterized a novel *Brucella* sp. from a dog presenting with orchiepididymitis in Costa Rica. The isolate, BCCN84.3, displays smooth colonial morphology and clusters within the classical *Brucella* clade containing all the major pathogenic species. The genome contains all the genes required for virulence but is distinct from the classical rough species *Brucella canis*, associated with brucellosis in dogs. The authors fall short of assigning a taxonomic name, citing current difficulties around the ongoing debate of the *Brucella* species concept, but raising awareness of this group will allow its significance, dispersal and zoonotic potential to be explored. With similar regard to the theme of extending ecological niches Jaý et al. identified an isolate similar to *Brucella microti*—a species originally described in voles but since found in wild boar, foxes and persisting in soil—in a domestic marsh frog in France. While many *Brucella inopinata*-like strains of the genetically atypical *Brucella* group have been associated with various species of tropical frogs recently (Scholz et al., 2016) this is the first finding of other species, closer to classical *Brucella* species, in amphibians.

## Molecular Typing, Evolution, Phylogeography

Several papers used molecular tools to explore *Brucella* diversity at the sub-species level particularly focusing on the major pathogenic species *Brucella melitensis* (ovine/caprine), *Brucella abortus* (bovine), and *Brucella suis* (porcine) The Topic included papers that used two of these approaches, multi-locus variable number of tandem repeat analysis (MLVA) and multi-locus sequence analysis (MLSA), to examine the global population structure of the genus. Whatmore et al. reported application of an extended MLSA tool to a globally and temporally diverse collection of >500 isolates, as well as the launch of an online database allowing the community to interrogate existing data and compare new profiles. Despite relatively low resolution, over 100 sequence types were identified by MLSA and new insights into the global evolutionary history of the genus were provided with evidence of the existence of lineages with restricted host or geographical ranges. Notably extensive previously undescribed genetic diversity was noted in African isolates with two early branching *B. abortus* clades appearing confined to Africa while other, later emerging, lineages have spread globally. A further important observation was the lack of congruence between biovar, long used as a crude epidemiological marker in these species, and genotype, particularly in *B. melitensis* but also to some extent in *B. abortus*. In a similar study Vergnaud et al. used MLVA to examine the population structure of a collection of >1,400 *Brucella* isolates from different animal and geographic origins isolated over three decades. In contrast to the situation in more rapidly evolving bacteria MLVA in *Brucella* provides useful resolution even at global/taxonomic levels as well as for local epidemiology, a point confirmed as authors highlighted the congruence of MLVA groupings with those reported in the above MLSA study. Once again work highlighted a lack of congruence between biovar and genotype and suggested that, particularly for *B. melitensis*, the biovar concept is of limited value and genome-based methods may be better placed to answer questions regarding epidemiology and tracking epidemic strains in the future.

In the topic MLVA was also applied in local situations to explore transmission pathways. Brucellosis is a serious public health problem in China (Jiang et al., 2020). Liu et al. characterized isolates of *B. melitensis* from Ulanqab using MLVA to suggest lack of control of animal movements or movement of contaminated food products between regions. In a larger study in Kazakhstan Shevtsova et al. examined >1,300 human derived isolates of *B. melitensis* showing that, as in the Liu et al. study above, isolates cluster within the previously identified “Eastern Mediterranean” group of *B. melitensis*. Both studies highlight once again the lack of any clear relationship between biovar and genotype in *B. melitensis*. Much less is known about *Brucella* diversity in Africa—Sanogo et al. reviewed MLVA types of *B. abortus* biovar 3 isolates in West Africa and highlighted the need for more studies to understand the epidemiology of brucellosis in this region. Foster et al. used MLVA, along with SNP assays, to identify a lineage of *B. melitensis* circulating in multiple animal species in Oman distinct from most other Middle Eastern isolates associated with endemic disease. The lineage appears most closely related to isolates from North Africa suggesting an African origin for some *B. melitensis* isolates in Oman likely through livestock trade. This study once again hinted at substantial under-sampled *Brucella* genetic diversity in Africa.

Cloeckaert et al. examined genes encoding Omp2 porins. These represent some of the most genetically diverse *Brucella* genes and were historically used as typing tools. Utilizing WGS data the authors propose an evolutionary pathway from two ancestral genes in genetically atypical *Brucella* through progressive genetic loss in *omp2b* on the path to the classic pathogenic species. Changes appear to reflect extensive recombination events with *omp2a* and were correlated to increasing sugar permeability of the porin that the authors speculate might be related to environmental adaptation to survive conditions on the pathway to the current status as intracellular pathogens.

Comparative genomics was applied by Holzapfel et al. to examine *B. melitensis* isolates from an outbreak infecting different hosts (human, bovine, and ovine) in order to identify evidence of host adaptation. Only a single SNP, and no genome rearrangements at all, were seen although the possibility of differences in expression in different hosts was not explored. Another important comparative genomic study provided a contrasting picture of genomic stability and highlights a common concern in microbiology—variability between reference strains assumed to represent a single common entity. *B. abortus* 2308 is a reference strain often used in virulence studies or as a challenge strain in vaccine trials. Suárez-Esquivel et al. compared the genomes of *B. abortus* 2308 stocks from three laboratories and revealed significant differences, particularly in indels related to insertional elements, suggesting that variability accumulated due to transposition events. The study challenges the idea that reference strains are non-changing entities in time and space and may question the validity of comparison between studies using the “same” reference strain.

## Phage Biology and Typing

Three papers focus on phage or phage resistance. Lytic phages have been used historically to type *Brucella* strains and Hammerl et al. sequenced known reference phage and, analogous to the situation described above, the same reference phage from different laboratories were not always genetically identical and may display differences in host range. The same study also analyzed 22 non-reference brucellaphages suggesting some new candidates that may be useful in diagnostics. Jäckel et al. also explored phage in the related *Ochrobactrum* group, showing for the first time that active prophages are common in this group. Li et al. examined a smooth phage resistant *B. abortus* strain isolated in China and compared it with phage sensitive strains. Although they failed to categorically identify a mechanism of phage resistance a number of indels and point mutations differentiating the strains were identified.

## *Brucella* Cell Envelope and Surface Structures

Three papers focus on the role and properties of lipopolysaccharide (LPS) and lipids of the *Brucella* cell envelope. *Brucella* LPS plays a major role in virulence impairing recognition by the innate immune system and delaying immune responses (Roop et al., 2021). The LPS core is a branched structure involved in resistance to complement and polycationic peptides. Mutants in glycosyltransferases required for the synthesis of the lateral branch not linked to the O-polysaccharide (O-PS) are attenuated, and have been proposed as vaccine candidates. The chemical structure of the *Brucella* LPS core suggests that, in addition to the already identified WadB and WadC glycosyltransferases, others could be implicated in the synthesis of this lateral branch. By screening *B. abortus* LPS mutants Salvador-Bescós et al. identified a new glycosyltransferase gene named *wadD*. A *wadD* mutant lost reactivity against antibodies that recognize the core section while keeping an intact O-PS. WadD glycosyltransferase may thus be involved in the addition of one or more sugars to the core lateral branch. Further experiments indicated its role in resistance to components of the innate immune system and in chronic stages of infection. These results corroborate and extend studies indicating that the *Brucella* LPS core is a branched structure that constitutes a steric impairment preventing elements of the innate immune system protecting against *Brucella*.

Conde-Álvarez et al. investigated *Brucella* homologs of *lptA, lpxE*, and *lpxO*, three genes that in some pathogens encode enzymes involved in masking the LPS pathogen-associated molecular pattern (PAMP), to avoid rapid recognition by innate immunity. They encode putative enzymes involved in lipid A or other lipids modification. In this study, despite their participation in cationic peptide resistance of *B. melitensis*, none of the *B. melitensis* mutants for the respective genes were attenuated in dendritic cells or mice, which suggest they are not significantly involved in alteration of the PAMP properties of *Brucella* LPS and free-lipids, and were not positively selected during the adaptation to intracellular life.

Smooth (S) LPS (S-LPS) is a major virulence factor in smooth *Brucella* species carrying an O-PS on the LPS core. The O-PS consists of an *N*-formyl-perosamine homopolymer and constitutes the major antigen in serodiagnostic tests. Martínez-Gómez et al. report that the *Brucella* O-PS can be structurally and antigenically modified using *wbdR*, the acetyl-transferase gene involved in *N*-acetyl-perosamine synthesis in *Escherichia coli* O157:H7. Introducing *wbdR* into the *Brucella* genome resulted in loss of the main *Brucella* O-PS immunodominant epitopes. *wbdR* constructs produced chronic infections in mice and triggered antibodies to new immunogenic epitope(s) that can be differentiated from those evoked by the wild-type strain in S-LPS ELISAs. These results raise the possibility of developing vaccines that are both antigenically tagged, and lack diagnostic epitopes of virulent field strains, thereby solving the diagnostic interference created by current *Brucella* vaccines.

A fourth paper focuses on the cell envelope of *Brucella ovis*, a non-zoonotic species lacking a specific vaccine. *B. ovis* presents a narrow host range (rams), a unique biology relative to other *Brucella* species, and important distinct surface properties. To develop a specific vaccine, Sidhu-Muñoz et al. investigated multiple mutants for nine cell-envelope-related genes. Among several combinations of mutations investigated in a mouse virulence model, a *B. ovis* mutant deleted for three genes (*omp10, ugpB*, and *omp31*) appeared an interesting vaccine candidate. It showed similar infectious behavior to the wild-type strain up until week 3 post-infection, but was then totally cleared from the spleen. In mice protection assays, in comparison to the live attenuated *B. melitensis* Rev1 reference vaccine, the triple mutant induced limited splenomegaly, a significantly higher antibody response against whole *B. ovis* cells, and better protection against challenge with a virulent *B. ovis* strain. As this vaccine candidate lacks Omp31, a highly immunogenic *B. ovis* protein, differentiation between infected and vaccinated animals may also be possible.

## Intracellular Lifestyle

Six papers in the topic focus on factors important in the intracellular lifestyle of *Brucella*. Rossetti et al. analyzed the *in vivo* temporal transcriptional profile of *B. melitensis* during the initial 4 h interaction with cattle. It revealed that *in vivo Brucella* sense and actively regulate their metabolism through transition to an intracellular lifestyle. Other *Brucella* pathways involved in virulence, such as ABC transporters and T4SS, were repressed suggesting a silencing strategy to avoid stimulation of the innate immune response very early in the infection process. It supports the idea that *Brucella* employ a stealthy strategy at the onset of infection. Further, using other approaches, unanticipated interactions were identified suggestive of new virulence factors and mechanisms responsible for evasion of the immune response.

Live attenuated *B. melitensis* Rev.1 vaccine is widely used to control small ruminant brucellosis. Following uptake by the host cell, *Brucella* replicate inside a membrane-bound compartment whose acidification is essential for pathogen survival. By comparative transcriptome analysis of Rev.1 and virulent strain 16M, Salmon-Divon et al. identified 403 genes responding differently to acidic conditions in the two strains, consisting of genes involved in crucial cellular processes, including metabolic, biosynthetic, and transport processes. A number of genes downregulated in Rev.1 under acidic conditions were identified, possibly explaining the attenuated virulence of Rev.1.

The microtubule (MT) cytoskeleton regulates several cellular processes related to the host immune system. Alves Silva et al. used nocodazole to induce MTs depolymerization and paclitaxel or recombinant (r) TIR (Toll/interleukin-1 receptor) domain containing protein (TcpB) to induce MT stabilization in bone marrow-derived macrophages infected with *B. abortus*. In this infection model, intracellular trafficking and maturation of *Brucella*-containing vesicles (BCVs) appeared affected by partial destabilization or stabilization of the MTs network. Complementary macrophage infection experiments indicated that modulation of MTs affects crucial steps of *B. abortus* pathogenesis, including BCV maturation, intracellular survival and IL-12 secretion in infected macrophages.

Some *Brucella* isolates require increased CO_2_ for growth, especially on initial isolation. By comparing differences in gene content among different CO_2_-dependent and CO_2_-independent *Brucella* strains, Garcia Lobo et al. confirmed that carbonic anhydrase (CA) II is the enzyme responsible for this phenotype. *Brucella* species contain two CAs of the β family, CA I and CA II. Genetic polymorphisms were identified for both of them in different isolates, but only those putatively affecting the activity of CA II correlated with the CO_2_ requirement of the corresponding isolate. CO_2_-independent mutants arise easily *in vitro*, and carry compensatory mutations that produce a functional full-length CA II. Unlike *in vitro* growth, growth *in vivo* in a mouse model of infection provided a significant advantage to the CO_2_-dependent strain. This could explain why some *Brucella* isolates are CO_2_-dependent in primary isolation.

Several genes associated with intracellular trafficking and multiplication have been identified in *Brucella* spp. However, the sophisticated post-transcriptional regulation and coordination of gene expression that enable adaptation to changes in environment and evasion of host cell defenses are not fully understood. Bacteria small RNAs (sRNAs) play a significant role in post-transcriptional regulation in a number of bacteria. Dong et al. identified several sRNAs in *Brucella* spp., and found that over-expression of a sRNA, termed BASI74, led to alteration in virulence of *Brucella* in a macrophage infection model. The expression of BASI74 increased when *B. abortus* was grown in acidic media. Four genes were identified as targets of BASI74. Among them, BABI1154, was predicted to encode a cytosine-N4-specific DNA methyltransferase, which protects cellular DNA from the restriction endonuclease in *Brucella*. BASI74 thus plays an important role in *Brucella* survival in a macrophage infection model, speculatively by its connection with stress responses or impact on restriction-modification systems.

The best-characterized brucellae infect livestock, behaving as stealthy facultative intracellular parasites. This stealthiness depends on envelope molecules with reduced PAMPs, as revealed by low lethality and the ability to persist in mice of these bacteria. Infected cells are often engorged with brucellae without signs of distress, suggesting that stealthiness could also reflect an adaptation of the parasite metabolism to use local nutrients without harming the cell. Zúñiga-Ripa et al. compared key metabolic abilities of virulent *B. abortus* 2308 and *B. suis* strain 513, representative of the ancestral biovar 5 found in wild rodents. Strain 513 used a larger number of C substrates and showed faster growth *in vitro*, two features similar to those of *B. microti*, a species phylogenomically close to strain 513 infecting voles. However, whereas *B. microti* shows enhanced lethality and reduced persistence in mice, strain 513 was similar to *B. abortus* 2308 in this regard. Further analyses showed similarities and discrepancies in metabolic pathways of the strains studied that may reflect a progressive adaptation of brucellae to intracellular growth.

## Immune and Cellular Responses

Establishment of a Th1-mediated immune response characterized by the production of IL-12 and IFNγ is essential to control brucellosis. Leukotrienes derived from arachidonic acid (AA) metabolism negatively regulate a protective Th1 immune response against bacterial infections. Gagnaire et al. demonstrated that *B. abortus* strongly stimulates the prostaglandin (PG) pathway in dendritic cells (DC). They also showed induction of AA production by infected cells correlated with expression of *Ptgs2*, encoding the downstream cyclooxygenase-2 (COX-2) enzyme in infected DC. By comparing infection routes (oral, intradermal, intranasal, and conjunctival), the intradermal inoculation route was identified as the more potent in inducing *Ptgs2* expression but also in inducing a local inflammatory response in the draining cervical lymph nodes (CLN). NS-398, a specific inhibitor of COX-2 enzymatic activity decreased *B. melitensis* burden in the CLN after intradermal infection. This effect was accompanied by a decrease of IL-10 and a concomitant increase of I*fng* expression. Altogether, these results suggest *Brucella* has evolved to take advantage of the PG pathway in the harsh environment of the CLN to persist and to subvert immune responses. This work also proposed that novel strategies to control brucellosis may include the use of COX-2 inhibitors.

Giambartolomei et al. reviewed mechanisms of osteoarticular brucellosis, a common presentation of human disease. The three commonest forms of osteoarticular involvement are sacroiliitis, spondylitis, and peripheral arthritis. *B. abortus* induces bone damage through diverse mechanisms where TNF-α and the receptor activator of nuclear factor kappa-B ligand, the natural modulator of bone homeostasis, are involved. These processes are driven by inflammatory cells. In addition, *B. abortus* has a direct effect on osteoarticular cells and tilts homeostatic bone remodeling. These bacteria inhibit bone matrix deposition by osteoblasts, and modify the phenotype of these cells to produce matrix metalloproteinases and cytokine secretion, contributing to bone matrix degradation. *B. abortus* also affects osteoclasts by inducing osteoclastogenesis and osteoclast activation; thus, increasing mineral and organic bone matrix resorption, contributing to bone damage. Pathology induced by *Brucella* also involves joint tissue and analysis of *B. abortus*-infected synoviocytes indicated bacteria also replicate in their reticulum indicating use of this cell type for intracellular replication during the osteoarticular localization of the disease.

## Genital Tropism

Erythritol is the preferential carbon source for most brucellae. Since this polyol is abundant in genital organs of ruminants and swine, it is accepted that erythritol accounts, at least partially, for the characteristic genital tropism of brucellae. Nevertheless, proof of erythritol availability and essentiality during *Brucella* intracellular multiplication has remained elusive. A study by Barbier et al., using wild-type *B. abortus* and erythritol utilization mutants, showed that erythritol was available, but not required, for *B. abortus* multiplication in bovine trophoblasts. Mice and humans have been considered to lack erythritol but this study showed it is available, but not required, for multiplication in human and murine trophoblastic and macrophage-like cells, and in mouse spleen and conceptus. These results led to the hypothesis that there may be erythritol in tissues of mammals other than ungulates, with evidence for the involvement of the host aldose reductase, an enzyme that can catalyze the synthesis of polyols including erythritol.

In a linked opinion article Letesson et al. noted the coincidence between the possible presence of lactate, glutamate, and glycerol in the placenta and male genital organs and fluids, favored habitats of brucellae, and nutritional requirements of *Brucella* identified in classical studies performed many decades ago (Gerhardt et al., 1950).

## Conclusion

In conclusion, this Research Topic provides a comprehensive and up-to-date summary of multiple aspects of *Brucella* pathogens, from their taxonomic position within the family *Brucellaceae* to their main pathogenic mechanisms and induced immune responses.

## Author Contributions

All authors listed have made a substantial, direct and intellectual contribution to the work, and approved it for publication.

## Conflict of Interest

The authors declare that the research was conducted in the absence of any commercial or financial relationships that could be construed as a potential conflict of interest.
